# Applying modern measurement approaches to constructs relevant to evidence-based practice among Canadian physical and occupational therapists

**DOI:** 10.1186/s13012-018-0844-4

**Published:** 2018-12-18

**Authors:** Fadi Al Zoubi, Nancy Mayo, Annie Rochette, Aliki Thomas

**Affiliations:** 1 McGill University, Montreal, QC Canada; 2 Centre for Interdisciplinary Research in Rehabilitation of Greater Montreal, Montreal, QC Canada; 3 Center for Outcomes Research and Evaluation (CORE), Research Institute of McGill University Health Center, Montreal, QC Canada; 4 Université de Montréal, Montreal, QC Canada; 5 Centre for Medical Education, Montreal, Canada

**Keywords:** Rasch Measurement Theory, Evidence-based practice, Self-efficacy, Attitudes, Knowledge, Resources, Measure, Measurement, Rehabilitation, Physical therapy, Occupational therapy

## Abstract

**Background:**

Evidence-based practice (EBP) is a complex process. To quantify it, one has to also consider individual and contextual factors using multiple measures. Modern measurement approaches are available to optimize the measurement of complex constructs. This study aimed to develop a robust measurement approach for constructs around EBP including practice, individual (e.g. knowledge, attitudes, confidence, behaviours), and contextual factors (e.g. resources).

**Methods:**

One hundred eighty-one items arising from 5 validated EBP measures were subjected to an item analysis. Nominal group technique was used to arrive at a consensus about the content relevance of each item. Baseline questionnaire responses from a longitudinal study of the evolution of EBP in 128 new graduates of Canadian physical and occupational therapy programmes were analysed. Principles of Rasch Measurement Theory were applied to identify challenges with threshold ordering, item and person fit to the Rasch model, unidimensionality, local independence, and differential item functioning (DIF).

**Results:**

The nominal group technique identified 70/181 items, and modified Delphi approach identified 68 items that fit a formative model (2 related EBP domains: self-use of EBP (9 items) and EBP activities (7 items)) or a reflective model (4 related EBP domains: attitudes towards EBP (17 items), self-efficacy (9 items), knowledge (11 items) and resources (15 items)). Rasch analysis provided a single score for reflective construct. Among attitudes items, 65% (11/17) fit the Rasch model, item difficulties ranged from − 7.51 to logits (least difficult) to + 5.04 logits (most difficult), and person separation index (PSI) = 0.63. Among self-efficacy items, 89% (8/9) fit the Rasch model, item difficulties ranged from − 3.70 to + 4.91, and PSI = 0.80. Among knowledge items, 82% (9/11) fit the Rasch model, item difficulties ranged from − 7.85 to 4.50, and PSI = 0.81. Among resources items, 87% (13/15) fit the Rasch model, item difficulties ranged from − 3.38 to 2.86, and PSI = 0.86. DIF occurred in 2 constructs: attitudes (1 by profession and 2 by language) and knowledge (1 by language and 2 by profession) arising from poor wording in the original version leading to poor translation.

**Conclusions:**

Rasch Measurement Theory was applied to develop a valid and reliable measure of EBP. Further modifications to the items can be done for subsequent waves of the survey.

**Electronic supplementary material:**

The online version of this article (10.1186/s13012-018-0844-4) contains supplementary material, which is available to authorized users.

## Background

Health care professionals are expected to integrate best available research evidence, patients’ preferences, and their clinical expertise to support clinical decision-making, a process known as evidence-based practice (EBP) [1]. Production of high-quality research in fields related to rehabilitation over the past 15 years [2] has provided evidence for occupational therapists (OTs) and physical therapists (PTs) to guide practice [3–5]. As a result of this exponential rise in knowledge, there is an urgent need to mobilize evidence into clinical practice [6]. According to the World Health Organization (WHO), rehabilitation is defined as “a set of interventions designed to optimize functioning and reduce disability in individuals with health conditions in interaction with their environment” [7]. Without implementing effective interventions, rehabilitation will not be successful [8–11]. As a response to these expectations [12, 13], a priority for all professional OT and PT programmes was to emphasize EBP. Despite the emphasis on teaching and promoting the competencies associated with EBP in professional programmes, measuring EBP remains a daunting challenge.

EBP is a complex area of enquiry necessitating multiple measurement approaches to identify the practice itself and the individual and contextual factors influencing it. Several socio-cognitive theories have been applied in research to identify and tackle these factors [14–16]. To determine if EBP is changing professional practices, improving the quality of care, and informing organizations [17], there is a need to administer several multi-item questionnaires covering the relevant domains [18]. The use of validated and reliable measures of EBP outcomes is essential to improve EBP across studies and to inform areas for contextual change [17].

### Measurement challenges

There are over 100 tools to measure domains related to EBP [19–22]. As a result, many items cover the same construct but with different phrasing and response options. There is always a need for parsimony in measurement, as redundancy can be a reason for non-completion and can produce false reliability [23]. The removal of redundant items from the questionnaire can raise concerns for fear of invalidating the interpretation of the total score derived from the original set of items. However, there is a vast literature on the validity of total scores derived from ordinal measures [24]. Typically, summing the numerical labels assigned to each ordinal response option is done to produce a total score. This score does not necessarily have mathematical properties nor does every item contribute equally to the total. Application of Rasch Measurement Theory (RMT) can shed light on how individual items contribute to a theoretically defined construct used to form a measure [25]. The basis of RMT is the Rasch model, named for Georg Rasch the Danish statistician, which is a probabilistic model used to situate a person and an item on a linear continuum from least able (easiest item) to most able (hardest item) [25].

RMT is one of the several measurement theories that have been applied in the context of health care. The best-known theory is Classical Test Theory which assumes that the true score is a function of the total score plus error; the error is assumed to be the same for each person and for each item [26]. Theories that are based on how each item behaves with respect to other items in the theoretical construct and how the items align along an expected linear hierarchy, from easiest to hardest, do not assume that these errors are the same. Therefore, the location and error for each item and each person are estimated. The location of each item along this “ability” continuum is estimated by a logit transformation of participants’ responses to each level of each item. An item response category that 50% of participants endorse, the middle item, has a logit of 0. The optimal linear scale for the items is required to have a mean of 0 and a standard deviation of 1, with locations all along the continuum from − 4 to + 4 logits. This represents the theoretical range of a standard normal distribution with a mean of 0 and a standard deviation of 1. The person’s values along this continuum are also optimal when they follow this standard normal distribution.

RMT has a number of requirements including measuring only one construct (i.e. unidimensionality). The analytical details of how to apply and interpret Rasch analysis to a set of person responses to items are presented in Table 1.

**Table 1:** Explanation of steps taken to fit the data to the Rasch model

Threshold order	There should be a logical ordering to the response options such that endorsing a more optimal response option should situate the person at a higher level of the latent trait. That means a person with higher ability (for example a knowledgeable clinician in EBP) is expected to select higher response options on an ordinal scale. At lower ability, more clinicians should endorse a lower response level, and fewer should endorse a higher response level. If the thresholds are disordered, the response options need to be rescored, sometimes reducing the responses to binary. The number of thresholds is equal to the number of response options - 1 and reflects the number of “jumps” the person has to make for each item.
Fit to the Rasch model	The items should line up hierarchically such that those items that need little ability to endorse at the most optimal response level are at the low end and those items requiring more ability to endorse are higher. Overall goodness of model fit is indicated by a non-significant chi-square test (*p* > 0.05) after a Bonferroni adjustment for the number of items. The fit of each item and each person is as important, or even more important, than overall fit. Item and person fit is indicated when fit residual (deviance from pure linearity) values are within ± 2.5 and the chi-square test for fit is non-significant (> 0.05). Those items that fail this criterion need to be looked at carefully to ensure their importance in scoring the latent trait. A fit residual of greater than + 2.5 indicates the item does not fit the latent trait; a fit residual of less than − 2.5 indicates the item overfits and may be redundant.
Unidimensionality	A requirement of the Rasch model is that a single latent trait is being measured. This is assessed using a principal component analysis (PCA) of the fit residuals. The person-ability estimates derived from all pair-wise comparisons of the two most disparate set of items (those with the highest positive and negative loadings on the first factor) are compared using independent *t* tests. For a set of items to be considered unidimensional, less than 5% of *t* values should be outside ± 1.96. When this value is greater than 5%, a binomial test of proportions is used to calculate the 95% confidence interval (CI) around the *t* test estimate. Evidence of unidimensionality is still supported if the 5% value falls within the 95%CI.
Response dependency	The uniqueness of the information provided by the items is a requirement of the Rasch model. Items with pair-wise residual (after controlling for the latent trait) correlations greater than 0.3 could indicate lack of independence of the responses which inflates the reliability. Solutions include creating a super-item which combines the response options across items or choosing the one item that best suits the testing context.
Differential item functioning (DIF)	The items should have the same ordering of difficulty across all people being measured defined by personal factors such as in this study, PT or OT, gender, and language. DIF is an indicator of item bias. Typically, DIF is indicated with a significant *F* test from a two-way analysis of variance. A caution is that with large and sample sizes, anything may be significant; with small sample sizes, nothing may be significant. A close visual inspection of the item characteristic curve plotted by the level of each factor will support or not the information from the statistical approach. Two options are available for items with DIF, deletion or split scoring.
Targeting	An ideally targeted measure should include a set of items that spans the full range of the theoretical latent construct (− 4 to + 4 logits) and have a mean location of 0 with a standard deviation (SD) of 1. Ideally, the person estimates from this measure should be centred on location 0 with a SD of 1.
Discrimination or person separation	This indicates how well people are differentiated by the spread of the item difficulty. The person separation index (PSI) is interpreted like a Cronbach’s alpha. The larger the index, the better is the discrimination which facilitates the measurement of change. Values of > 0.9 are suitable for measuring within-person change; values > 0.7 are suitable for detecting group differences.

Items that do not fit the Rasch model should not be used in the total score until improved. Only few EBP measures [27–32] have used Rasch analysis for developing the scoring system, and none of these items were designed for rehabilitation EBP. Moreover, none of these studies were designed for Canadian professionals where the items have to be both in English and French.

### Study context

In the context of a study on the evolution of individual characteristics (including knowledge, attitudes, confidence), contextual factors (including support for work setting), and actual use of EBP in graduates of the professional M.Sc. programme in PT and OT (grant number 148544) [33], 5 existing measures were assembled to tap into these important EBP constructs. This resulted in 181 items which clearly would be a barrier to study recruitment and completion. In addition, many of the items were redundant leading to low efficiency [34] or had multiple concepts in one item leading to high content density [35]. An example of item redundancy is the “feedback process” subcategory of the Alberta Context Tool [36] that includes 5 items that can be reflected in one. These items (*I routinely receive information on my team’s performance on data like the examples provided above, our team routinely discuss this data informally, our team has a scheduled formal process for discussing this data, our team routinely formulates action plans based on the data, our team routinely compares our performance with others*) can be captured in one item (*our team routinely monitors our performance with respect to the action plans*). Another example of multiple concepts in one item exists in the subcategory “sympathy” in the Evidence-Based Practice Profile Questionnaire-2 which is *Critical appraisal of the literature and its relevance to the client is not very practical in the real world of my profession*. This item fits in both the “sympathy” and “relevance” subcategories. These shortfalls in item development can lead to inconsistency in responses and biased estimates of change. From a measurement perspective, there is value in the items from multiple questionnaires as they could be considered to form a pool of items from which new combinations could be constructed with legitimate total scores.

Therefore, the global aim of this study was to apply a robust measurement approach on constructs around EBP including practice, individual characteristics variants (e.g. knowledge, attitudes, confidence, behaviours), and contextual factors (e.g. resources). The specific study objectives were to (i) identify the extent to which items from existing EBP measures reflect constructs suitable for use in a survey of EBP in PTs and OTs and (ii) the extent to which the items reflective of EBP constructs fit the expected unidimensional hierarchy sufficient to create a total score with interval-like properties optimizing the estimation of change or differences across groups.

## Methods

### Study design

This study was based on three steps: a nominal group process [37], a modified Delphi approach [38], and a cross-sectional electronic survey. An item analysis was conducted on all items arising from the five EBP measures chosen for inclusion in the longitudinal study of the evolution of EBP in new graduates of Canadian PT and OT programmes mentioned above. Data from the newest PT/OT cohort to enter clinical practice were analysed to refine the measurement strategy for future phases.

### Population

The target population for the cross-sectional survey was all graduates (*n* = 1703) of the 28 Canadian OT and PT programmes that completed their professional education during the 2016–2017 academic year. To ensure complete ascertainment of graduates, participants were identified from university academic programmes. For the item analysis, the newest entry cohort was queried within 1-month post-graduation. The new graduates were prioritized for recruitment in order to assess EBP-related constructs at entry to practice and then subsequently over time. At the time of analysis, data from new graduates from 12 of the planned 28 university programmes were available.

### Measurement

To identify potential measures for the broader study, all relevant EBP measures reported in the literature were identified. Table 2 lists the 5 measures used in this study from which the 181 items were chosen by the research team as targeting the constructs of self-use of EBP and EBP activities, individual factors (attitudes towards EBP, confidence in applying EBP, knowledge), and contextual factors (specifically resources).

**Table 2:** Measures—description and psychometric properties

Original measure	Description	New measures	Items
EBPQ^2^ [39] Practice subscale	74 items; 5-point Likert Scale Time frame: past 6 months Psychometric data: acceptable internal consistency (Cronbach’s alpha (*α*) 0.85), test-retest reliability (intraclass correlation coefficient (ICC) 0.83 and convergent validity 0.66 [40].	Self-use of EBP: It was a term we chose to reflect actual application of EBP concepts, tools, and procedures into specific actions such as identifying knowledge related to a patient situation or the ability to formulate a research question to guide a literature search based on this gap.	9 items on a 5-point scale ranging from “never” to “more than 10 times a month”
		EBP activities: It can be defined as the implementation of research evidence to the surrounding environment such as in/formally shared and discussed literature/research findings with colleagues at work or patients.	7 items on a 5-point scale ranging from “never” to “daily”
EBPAS [41]	50 items; 5-point ordinal scale^§^ Psychometric data: acceptable good internal consistency (Cronbach’s *α* 0.90–0.93), good reliability (ICC 0.83), and convergent validity 0.66 [41–43].	Attitudes towards EBP	17 items on a 5-point scale ranging from “strongly disagree” to “strongly agree”
EPIC [44]	11 items; confidence from 0 to 100% Psychometric data: validated for both PTs and OTs. It has almost excellent internal consistency (Cronbach’s *α* 0.89) [44]. The ICC for test-retest reliability was 0.89 for PTs [45] and 0.92 for OTs [46].	EBP self-efficacy	9 items on a scale from 0 to 10 representing 0–100%
EBPQ [40]	24 items; 7-point Likert Scale Psychometric data: acceptable internal consistency (Cronbach’s *α* 0.91) and excellent test-retest reliability (ICC 0.94) [47].	Knowledge of EBP	11 items on a 5-point scale ranging from “never heard the term” to “understand and could explain to others”
ACT [36]	57 items; 5-point Likert Scale Psychometric data: acceptable internal consistency (Cronbach’s *α* > 0.80) [48].	EBP resources: It can be defined as the available resources at the workplace that allow clinicians to access and use EBP or encourage the clinicians to use EBP such as receiving recognition from manager/supervisor and workplace/college support the best practice.	17 items on a 5-point scale ranging from “strongly disagree” to “strongly agree”

*EBPQ* Evidence-Based Practice Profile Questionnaire, *EBPQ*^*2*^ Evidence-Based Practice Profile Questionnaire-2, *EBPAS* Evidence-Based Practice Attitude Scale, *EPIC* Evidence-Based Practice Confidence Scale, *ACT* Alberta Context Tool

^§^We used only 15 items since some of these items are repetitive in other measures

### Procedures

**Fig. 1:**
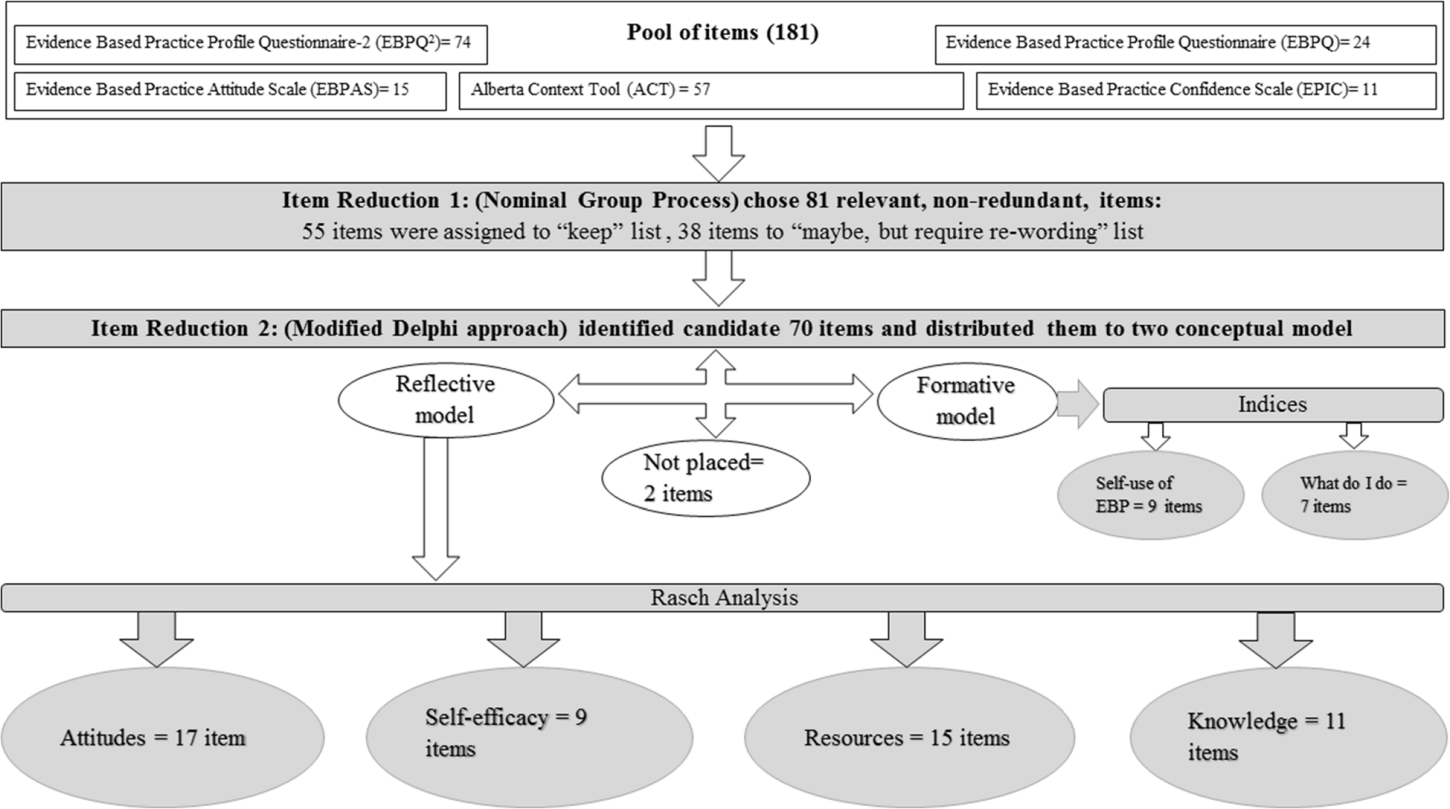
The procedures for appraising the items and identifying potential constructs

The procedures for appraising the 181 items and identifying potential constructs for further analyses are shown in Fig. 1. We used a three-phase process with two groups of experts (potential future respondents (user panel) and methodological (expert panel)) for the item analysis and then translated the results to French. The user panel comprised 12 PTs and OTs all with experience in clinical practice and training in EBP. The participants had a range of experience that covered the scope of practice of PT and OT including recent graduates and experienced clinician-researchers. The expert panel comprised the core research team members (AT, NEM, FAZ, AR).

#### Phase 1: Nominal group process

A nominal group process [36], involving both the user and expert panels, was used to screen each item by applying three criteria. Contextual relevance was defined based on whether the item was judged (by 80% or more of the user panel) to be relevant to the EBP and research/clinical scenarios facing PTs and/or OTs. Redundancy was to be avoided, and so, items were excluded if an item had a similar meaning to another item. In case of repetition, the item judged by > 50% of the user panel to have the clearest wording was retained. Re-wording was needed for items created for other health care providers such as doctors or nurses but relevant for EBP in our context. The expert panel suggested alternatives for re-wording the item, and the final decision was made by consensus (see the Additional file 1). This process reduced the initial 181 items to a pool of 81 items.

A modified Delphi process involving all the investigators on the research team (*n* = 8) was used to refine the items.

##### First round

An invitation e-mail was sent enclosing an explanation of the aim of the work and a description of the previous steps that had led to the generation of the item pool. Once the invitee accepted to participate, the item pool was sent by e-mail with specific instructions. Each investigator was asked to vote by marking on an Excel sheet an X under the category Item Clarity (completely clear or completely unclear) and under the category Informative (highly informative, moderately informative, not informative, not sure). Another category was created for additional comments. In this round, the goal was to clarify any redundancy or comprehension problems regarding each item [49]. Response frequencies for each item were calculated, and the experts’ identity was anonymized by a research assistant. Each item required 80% agreement from the panel in order to keep or remove the item as suggested by Lynn [49]. If the item showed < 80% agreement and there were comments for re-wording, then the item was kept to be re-worded and re-administered in the next round. All the responses were assigned to one of the three categories: keep without changes, keep with re-wording, and remove because of redundancy. At the end of this round, the expert panel met and re-worded the items that fell under the category of “re-word”. In case of any ambiguous comments, the investigator was contacted to seek clarifications for the comment. After reviewing all items, a final list was prepared for the second round.

##### Second round

An e-mail was sent to the expert panel for a final review and feedback on the changes applied to the items using similar procedures for rating as described in the first round.

This process resulted in 70 endorsed items from the pool of 81 items.

#### Phase 2: Meeting with the core team

Once all comments were in, two meetings with the core research team were held to identify the ideal set of items that would be carried forward in subsequent phases. Four activities were carried out. The first was to confirm the constructs and their labels. Second, items were assigned to constructs or left unassigned. Third, each item was prioritized for inclusion according to its relevance to the construct and whether the item indicated a high or low degree of EBP. The fourth activity was to consider whether the constructs fit best with a formative or reflective conceptual model. Formative models are where the items form the construct rather than reflecting it. For example, a priori, self-use of EBP practices was considered formative as there was a list of recommended practices. The more practices, the higher the EBP use. The most valid method for creating a legitimate total score for formative constructs is to count the number of items at a particular level of expertise. The construct self-efficacy is a good example of a reflective model, as having more self-efficacy or confidence in applying EBP in practice is reflected in people endorsing confidence in certain behaviours chosen to reflect the construct. A necessary but not sufficient criterion for a reflective model is that the items fit the underlying measurement model, here the Rasch model.

#### Phase 3: Translation

The quality of translation followed the *Guidelines for the Process of Cross-Cultural Properties of Self-Report Measures* [50]. This guideline helps the translation process into a new language through the following six steps: (1) initial (forward) translation—two professional translators with French-first language as their mother tongue (one was informed about the project, and the other translator was novice to the area) independently translated the final list of items from English to Canadian French; (2) synthesis of the translation—the same two translators synthesized the results of the two translations by preparing a consensus translation in the Canadian French language with the help of a research assistant; (3) back translation—two professional translators with English-first language as their mother tongue that are naive to measurement made back-translations; (4) use of an expert committee—in this step, a methodologist, PT, and OT professionals and a language professional met to come up with a clean version of the translation by solving any discrepancies through discussions until all the final items were judged to be linguistically equivalent; (5) testing the pre-final version—the clean version of the Canadian French set of items was completed by five clinicians and graduate students who were recent graduates (less than 5 years of clinical experience). This group identified some items to be hard to answer for recent graduates who have not worked in clinical practice. That led to suggest adding “My work does not involve clinical care” as a response option to all questions except those in knowledge or confidence domain; and (6) appraisal of the adaptation process—after adding “My work does not involve clinical care” as a response option, the final set of items was approved by the core team to be administered online.

### Rasch analysis

Rasch measurement analysis was carried out to test the fit of the items related to EBP to the Rasch model. Rasch model was considered fit by item/person if the observed item/person perform consistently like the expected item/person performance. This can be quantified using chi-square (*χ*^2^) probability value if the value is > 0.05 with a Bonferroni adjustment and item/person fit residuals (sum of person and item deviations) that were close to 0 with a standard deviation of 1. Item residual correlation matrix was examined for possible local item independence, and unidimensionality was explored using principal component analysis of the residuals. Individual item fit was evaluated using the *χ*^2^ probability value and the fit residual values. If *χ*^2^ probability value of < 0.05 (Bonferroni adjustment) and fit residual values ≥ 2.5, then the individual item was considered misfit [51, 52]. The person separation index (PSI) was used to assess the internal consistency reliability of the scale which is equivalent to Cronbach’s *α* [47]; however, it only uses the logit values instead of the raw scores [52], where a value of ≥ 0.7 was considered acceptable representing the minimum required to divide the participants into two distinct groups (low/high ability) [53, 54]. Differential item functioning (DIF) was assessed to identify items that work differently for some groups who have the same level of ability in the sample. DIF was tested by profession, gender, language, and the type of clinical setting. The items which did not fit theoretically or mathematically the Rasch model were removed. Items that showed DIF were either deleted or split. This step was repeated multiple times until all items fit the model and the measure was formed.

All Rasch measurement analyses were performed using the Rasch Unidimensional Measurement Model Software (RUMM) version 2030 [55]. All descriptive analyses were performed using the SAS statistical software (version 9.4) [56]. Data were reported as means ± standard deviation (SD) or as frequencies (percentages).

### Ethics

The research project from which the data were taken had ethical approval from all relevant university ethics committees to carry out the study.

## Results

The characteristics of the participants are shown in Table 3. Figure 1 shows the process and results of the item analysis. The nominal group process resulted in a streamlined group of 81 items, from the original pool of 181 items: 55 which could be used without modification (“keep” list) and 38 that would need some re-wording. For the modified Delphi approach, all 8 investigators participated in all rounds. For the first round, experts provided their ratings for all the items. For the additional comments, redundant ones having similar suggestion were grouped and reduced to produce 57 comments which included suggestions to rephrase the item or make it clearer. There was an agreement among the experts to keep without changes (51), keep with re-wording (29), and remove because of redundancy (11). For both the item clarity and informative agreement, initially, 41 items were rated to be completely clear and highly informative (> 80% agreement) while 29 items were rated to be completely unclear or moderately/not informative (< 80% agreement). The items that were rated to be completely clear were not necessarily rated as highly informative and the opposite way.

**Table 3:** Characteristics of graduates (*n* = 128)

Variable	Mean ± SD or *N* (%)
Age (years)	27.3 (6.9)
Gender	
Men/women/not answered	19/105/14 (14.9/82.9/3.1)
Language	
English/French	70/65 (51.9/48.1)
Degree	
PT/OT	53/75 (41.4/58.6)
Currently working	
Yes/no	73/55 (57.0/43.0)

For the second round, items were either kept as they were, removed, or re-worded in light of the experts’ comments. In total, 70 items were examined by the expert panel where no more major changes were required.

In total, 2 items were found to not be related to any domain resulting in a total of 68 items distributed on 6 EBP domains that were covered by the items. Two sets were considered to fit a formative model: self-use of EBP (9 items) and EBP activities (7 items). For these measures, the best total score is derived by counting the number of uses or activities carried out per day over the specified time frame.

### Self-use of EBP

For this construct, more frequent use of EBP does not necessarily translate to better EBP if every contact with a patient initiates an EBP activity. For example, identifying a gap in knowledge more than 10 times a month would seem problematic rather than desirable. For this measure, counting the number of practices used in the past 6 months would eliminate giving problematic behaviours more weight. A total score from 0 to 9 would now be the indicator for self-use of EBP (see Table 4).

**Table 4:** Results of analysis for self-use of EBP

Instructions: For each of the following activities, how often have you done the following in the past 6 months?: 5-point scale (Directives: Depuis 6 mois, à quelle fréquence avez-vous…)							
Item #	Description of item		Never	1 to 2 times	Almost every month	2 to 10 times a month	More than 10 times a month
1	E	Identify a gap in your knowledge related to a patient or client situation (e.g. history, assessment, treatment)?	0	1	1	1	1
	F	Cerner une lacune dans vos connaissances sur la situation d’un patient ou client (ex. antécédents, évaluation, traitement)?					
2	E	Formulate a question to guide a literature search based on a gap in your knowledge?	0	1	1	1	1
	F	Formuler une question pour orienter une recherche de la littérature fondée sur cette lacune dans vos connaissances?					
3	E	Effectively conduct an online literature search to address the question?	0	1	1	1	1
	F	Mener efficacement une recherche en ligne de la littérature pour tenter de répondre à mes questions?					
4	E	Critically appraise the strengths and weaknesses of study methods (e.g. appropriateness of study design, recruitment, data collection, and analysis)?	0	1	1	1	1
	F	Évaluer de manière critique les forces et faiblesses de certaines méthodes de recherche (ex. pertinence de la conception d’une étude, recrutement, collecte et analyse de données)?					
5	E	Critically appraise the measurement properties (e.g. reliability and validity, sensitivity and specificity) of standardized tests or assessment tools you are considering using in your practice?	0	1	1	1	1
	F	Évaluer de manière critique les caractéristiques de mesure (ex. fidélité et validité, sensibilité et spécificité) des tests normalisés ou des outils d’évaluation que vous pensez utiliser dans votre pratique?					
6	E	Interpret study results obtained using statistical tests and procedures (e.g. *t* tests, logistic regression?)	0	1	1	1	1
	F	Interpréter les résultats d’étude à l’aide d’outils et de procédures statistiques (ex. tests t, régression logistique)?					
7	E	Determine if evidence from the research literature applies to your patient’s/client’s situation?	0	1	1	1	1
	F	Déterminer si des preuves découlant d’une recherche de la littérature s’appliquent à la situation de votre patient ou client?					
8	E	Decide on an appropriate course of action based on integrating the research evidence, clinical judgment, and patient or client preferences?	0	1	1	1	1
	F	Décider d’un plan d’action approprié intégrant des données probantes, le jugement clinique et les préférences du client ou patient?					
9	E	Continually evaluate the effect of your course of action on your patient’s/client’s outcomes?	0	1	1	1	1
	F	Évaluer régulièrement les conséquences de votre plan d’action sur les résultats chez le patient ou client?					

0 = never; 1 = one time or more

*E* English, *F* French translation

### EBP activities

For EBP activities, more frequent use does indicate more EBP, and hence, a total number of activity days would be a reasonable metric. This requires assigning a frequency for each of the categories of never, monthly or less, bi-weekly, weekly, and daily over the past working month and giving the estimates of 0, 1, 2, 4, and 20 for these categories respectively. The total score is the cross-product of item days yielding activity days on a continuous scale (see Table 5).

**Table 5:** Results of analysis for the original items of the construct EBP activities

Instructions: In the past month, how often have you?: 5-point scale (Directives: Depuis un mois, à quelle fréquence avez-vous…)							
Item #	Description of item		Never	Monthly or less	Bi-weekly	Weekly	Daily
1	E	Integrated research evidence with your expertise	0	1	2	4	20
	F	Intégré des preuves découlant de recherches à votre expertise?					
2	E	Informally (e.g. outside of formal team or family meetings) shared and discussed literature/research findings with colleagues at work	0	1	2	4	20
	F	Partagé et discuté de manière informelle (ex. hors du cadre de réunions d’équipe ou de famille structurées) de résultats publiés ou de recherches avec des collègues au travail?					
3	E	Formally (e.g. during team or family meetings) shared and discussed literature/research findings with colleagues at work	0	1	2	4	20
	F	Partagé et discuté dans un cadre structuré de résultats publiés ou de recherches avec des collègues au travail?					
4	E	Shared and discussed literature/research findings with patients/clients	0	1	2	4	20
	F	Partagé et discuté de résultats publiés ou de recherches avec des patients ou clients?					
5	E	Read published research reports	0	1	2	4	20
	F	Lu des rapports de recherche publiés?					
6	E	Made time to read research	0	1	2	4	20
	F	Réservé du temps à la lecture de travaux de recherche?					
7	E	Attended in-services/workshops/courses in your organization?	0	1	2	4	20
	F	Assisté à des ateliers, séances de formation ou cours dans votre organisation?					

*E* English, *F* French translation

#### Conceptual model

**Fig. 2:**
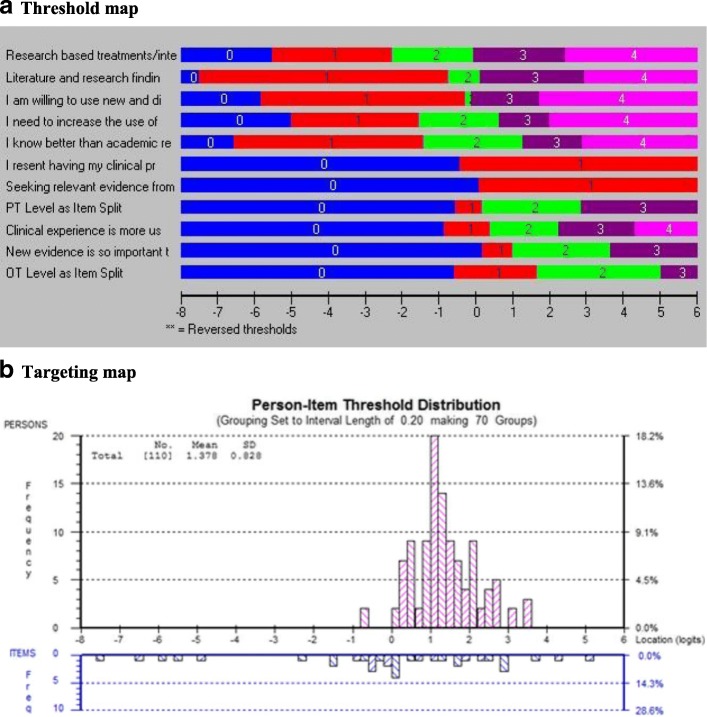
**a** Threshold map and **b** targeting map of “attitudes” construct

**Fig. 3:**
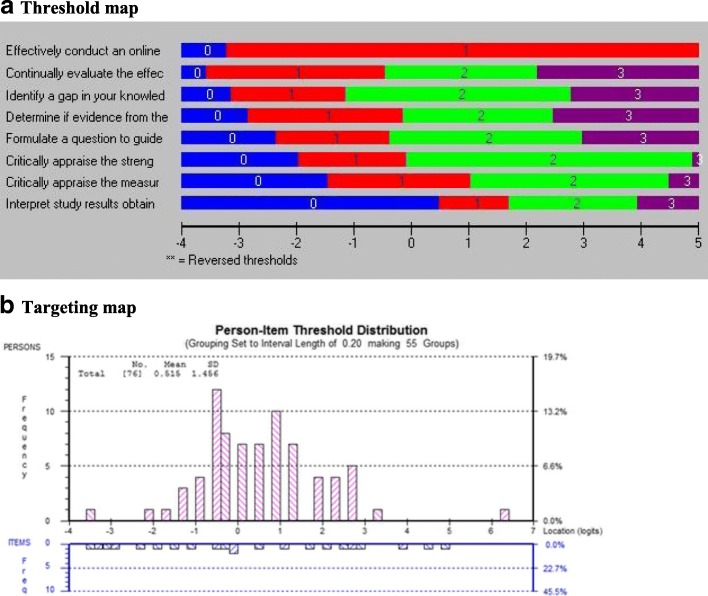
**a** Threshold map and **b** targeting map of “self-efficacy towards EBP” construct

**Fig. 4:**
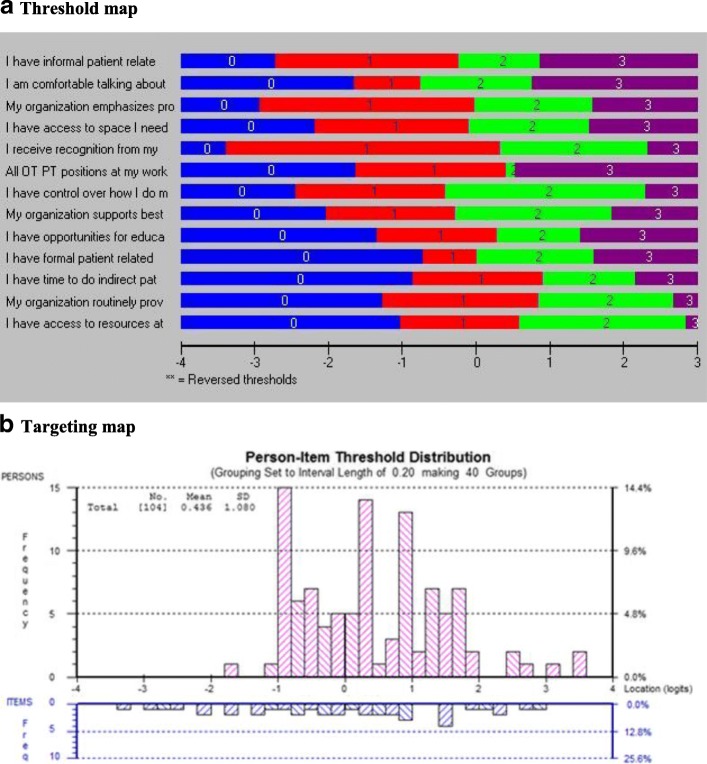
**a** Threshold map and **b** targeting map of “resources” construct

**Fig. 5:**
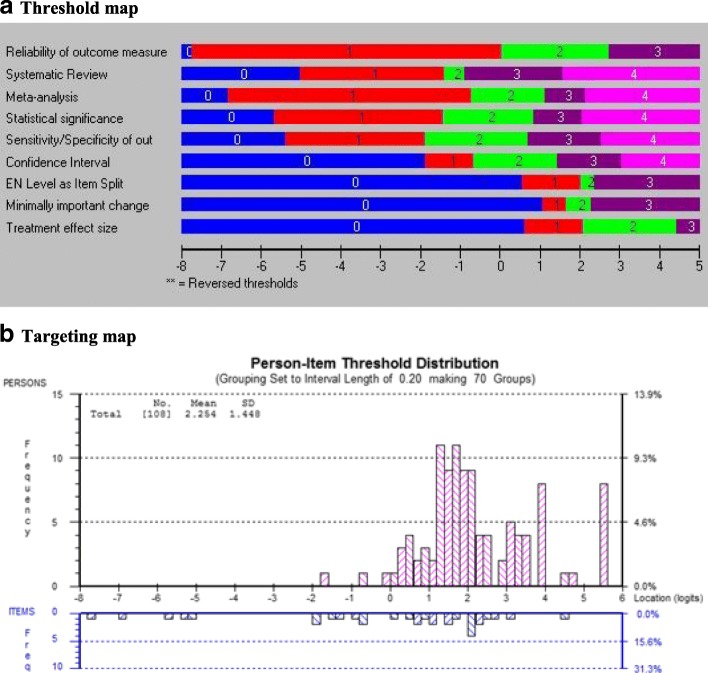
**a** Threshold map and **b** targeting map of “knowledge” construct

Four sets of items were considered to potentially fit a reflective conceptual model: attitudes towards EBP (*n* = 17 items), self-efficacy (*n* = 9 items), knowledge (*n* = 11 items), and resources (*n* = 15 items). Items that were compatible with a reflective model were tested to estimate the extent to which they fit the Rasch model, a necessary condition for a reflective model. Tables 4, 5, 6, and 7 and Figs. 2, 3, 4, and 5 present the results of the Rasch analysis for the constructs originally considered to be reflective.

**Table 6:** Results of Rasch Analysis for the Original Items of the Construct Attitudes towards EBP

Instructions: Please indicate your level of agreement with the following statements: 5-point Likert Scale (Directives: Veuillez indiquer à quel point vous êtes en accord avec les énoncés suivants)						
Item #	Description of Item		Response option rescored	Result		
				Item misfit	Local item dependency	DIF
1	E	New evidence is so important that I make the time in my work schedule	√	No	Yes	No
	F	(Les nouvelles données probantes sont tellement importantes que j’y consacre du temps dans mon horaire de travail				
2	E	My practice has changed because of evidence I have found	√	No	Yes	Profession (Item 2 split)
	F	Ma pratique a changé en raison de données probantes que j’ai découvertes				
3	E	Evidence based practice is fundamental to my professional practice	√	No	Yes with Item 5 (Item 3 deleted)	No
	F	La pratique fondée sur des données probantes est essentielle à l’exercice de ma profession				
4	E	I need to increase the use of evidence in my daily practice		No	No	No
	F	Je dois augmenter le recours aux données probantes dans ma pratique				
5	E	An evidence based practice approach improves the quality of my practice	√	No	No	Language (Item 5 deleted)
	F	Une pratique fondée sur des données probantes améliore la qualité de mon travail professionnel				
6	E	Literature and research findings are useful in my daily practice	√	No	Yes	No
	F	Les données tirées de la littérature et de la recherche sont utiles dans ma pratique de tous les jours				
7	E	Evidence based practice helps me to make decisions about patients/clients in my practice	√	No	Yes with Item 2 and 6 (Item 7 deleted)	No
	F	Le recours à des données probantes m’aide à prendre des décisions au sujet des patients ou clients de ma pratique				
8	E	I am willing to use new and different types of clinical interventions (e.g. assessment, treatment)developed by researchers to help my patients/clients	√	No	Yes	No
	F	J’accepterais de bon gré d’utiliser divers types d’interventions cliniques inédites (ex. évaluation, traitement) mises au point par des chercheurs pour aider mes patients ou clients				
9	E	I would try a new therapy/intervention even if it were very different from what I am used to doing	√	No	Yes with Item 8 (Item 9 deleted)	No
	F	J’essaierais un nouveau traitement ou une nouvelle approche même si elle était très différente de ce que je fais d’habitude				
10	E	I resent having my clinical practice questioned	√	No	No	No
	F	Je n’aime pas qu’on mette en doute ma pratique clinique				
11	E	I stick to tried and trusted methods in my practice rather than changing to anything new	√	No	No	Profession and language (Item 11 deleted)
	F	Dans ma pratique, j’adopte des méthodes fiables et éprouvées plutôt que de changer et essayer une nouvelle approche				
12	E	Clinical experience is the most reliable way to know what really works	√	No	Yes with Item 13 (Item 12 deleted)	No
	F	L’expérience clinique est la meilleure façon de voir ce qui fonctionne vraiment				
13	E	Clinical experience is more useful than scientific studies when I make decisions about my patients/clients	√	No	Yes	No
	F	L’expérience clinique est plus utile que les études scientifiques au moment de prendre des décisions au sujet de mes patients ou clients				
14	E	Critical appraisal of the literature is not very practical to do in my day-to-day practice	√	No	Yes with Item 15 (Item 14 deleted)	No
	F	Faire une évaluation critique de la littérature n’est pas très pratique dans ma pratique au jour le jour				
15	E	Seeking relevant evidence from scientific studies is not very practical in the real world	√	No	Yes	No
	F	Chercher des données probantes dans des études scientifiques n’est pas très pratique dans la réalité				
16	E	I know better than academic researchers how to care for my patients/clients	√	No	No	No
	F	Je sais mieux que des chercheurs théoriciens comment m’occuper de mes patients ou clients				
17	E	Research based treatments/interventions are not clinically useful	√	No	No	No
	F	Les traitements ou interventions découlant de la recherche ne sont pas utiles en clinique				

*DIF* differential item functioning, *E* English, *F* French translation

**Table 7:** Results of Rasch analysis for the original items of the construct self-efficacy towards EBP

Instructions: Please indicate how confident you are in your current level of ability by choosing the corresponding number on the following rating scale: 11-point continuous scale (Directives: Veuillez indiquer à quel point vous avez confiance en vos capacités actuelles en choisissant le nombre correspondant sur l’échelle d’appréciation suivante)						
Item #	Description of item		Response option rescored	Result		
				Item misfit	Local item dependency	DIF
1	E	Identify a gap in your knowledge related to a patient or client situation (e.g. history, assessment, treatment)?	✓	No	No	No
	F	Cerner une lacune dans vos connaissances sur la situation d’un patient ou client (ex. antécédents, évaluation, traitement)?				
2	E	Formulate a question to guide a literature search based on a gap in your knowledge?	✓	No	No	No
	F	Formuler une question pour orienter une recherche de la littérature fondée sur cette lacune dans vos connaissances?				
3	E	Effectively conduct an online literature search to address the question?	✓	No	No	No
	F	Mener efficacement une recherche en ligne de la littérature pour tenter de répondre à mes questions?				
4	E	Critically appraise the strengths and weaknesses of study methods (e.g. appropriateness of study design, recruitment, data collection, and analysis)?	✓	No	No	No
	F	Évaluer de manière critique les forces et faiblesses de certaines méthodes de recherche (ex. pertinence de la conception d’une étude, recrutement, collecte et analyse de données)?				
5	E	Critically appraise the measurement properties (e.g. reliability and validity, sensitivity and specificity) of standardized tests or assessment tools you are considering using in your practice?	✓	No	No	No
	F	Évaluer de manière critique les caractéristiques de mesure (ex. fidélité et validité, sensibilité et spécificité) des tests normalisés ou des outils d’évaluation que vous pensez utiliser dans votre pratique?				
6	E	Interpret study results obtained using statistical tests and procedures (e.g. *t* tests, logistic regression?)	✓	No	No	No
	F	Interpréter les résultats d’étude à l’aide d’outils et de procédures statistiques (ex. tests t, régression logistique)?				
7	E	Determine if evidence from the research literature applies to your patient’s/client’s situation?	✓	No	Yes	No
	F	Déterminer si des preuves découlant d’une recherche de la littérature s’appliquent à la situation de votre patient ou client?				
8	E	Decide on an appropriate course of action based on integrating the research evidence, clinical judgment, and patient or client preferences?	✓	No	Yes with items 7 and 9 (item 8 deleted)	No
	F	Décider d’un plan d’action approprié intégrant des données probantes, le jugement clinique et les préférences du client ou patient?				
9	E	Continually evaluate the effect of your course of action on your patient’s/client’s outcomes?	✓	No	Yes	No
	F	Évaluer régulièrement les conséquences de votre plan d’action sur les résultats chez le patient ou client?				

*DIF* differential item functioning, *E* English, *F* French translation

### Attitudes towards EBP items

The attitudes towards EBP items are shown in Table 6. Seventeen items were tested, all measured on a 5-point Likert Scale for the degree to which the respondent agrees or disagrees with the statement with an additional response options for “My work does not involve clinical care” in the event that the respondent does not provide hands-on direct care to patients but is involved in another aspects or rehabilitation practice (e.g. case management, clinical research). This latter option was merged with the “neutral” option. Nine items had disordered thresholds and needed rescoring. For 7 items, the categories “strongly disagree” and “disagree” were collapsed (items 1, 2, 3, 5, 7, 10, 11); for 3 items, the final rescoring resulted in only 2 (binary) categories (items 10, 14, 15). All 17 items fit the Rasch model after rescoring. Five items showed dependency with at least 1 other item, and the best-worded item was kept resulting in the deletion of items 3, 7, 9, 12, and 14. Three items showed DIF: item 2 showed DIF by profession, and the item was split to allow 2 different scoring; item 5 showed DIF by language, and as it was very close in content to item 3, it was deleted; and item 11 (*I stick to tried and trusted methods in my practice rather than changing to anything new*) showed DIF by language and profession. On close inspection, the English wording was idiomatic, and the French translation did not reflect the right meaning of the idiom. Item 13 is close in content, and so, item 11 was deleted. The final set of 10 items best reflecting the attitudes the EBP all fit the Rasch model and formed a measure (*χ*^2^ = 25.14, df = 22, *p* = 0.29). Figure 2 shows the threshold map (part a) and the targeting map (part b). The threshold map lists the items according to their average location on the latent trait (*x*-axis). The thresholds, transitions from 1 category of response to the next, are illustrated in different colours. There is a gradient across the items in the threshold location such that the easiest item, item 17 (see Table 6), is situated at the lowest end of the latent trait (− 7.51). The hardest item (item 2, split by profession) has the most difficult threshold at + 5.04. Figure 2b shows the location of the people and the items along the latent trait (*x*-axis) with the people above the line in pink and the items threshold below the line in blue. The items are poorly targeted to the sample primarily because the sample scored above 0, the expected mean location (mean 1.38; SD 0.83), whereas the range of items is from ≈ − 8.0 to + 5.0.

### Self-efficacy towards EBP

The self-efficacy towards EBP items are shown in Table 7 and Fig. 3. Nine items were tested; all measured on an 11-point scale from 0% (no confidence) to 100% (completely confident). All the items had disordered thresholds and needed rescoring. For eight items, the categories “90%” and “100%” were collapsed (items 1, 2, 4, 5, 6, 7, 8, 9); for one item, the final rescoring resulted in only two (binary) categories (item 3). For all items, the first four categories (0%, 10%, 20%, 30%) were merged; for all but item 3, the middle categories (40%, 50%, 60%) were collapsed. All nine items fit the Rasch model after rescoring. Item 8 showed dependency with two other items (items 7 and 9), and the best-worded items were kept resulting in the deletion of item 8. None of the items showed DIF. The final set of eight items best reflecting the self-efficacy towards EBP all fit the Rasch model and formed a measure (*χ*^2^ = 10.89, df = 16, *p* = 0.82). Figure 2a shows the gradient across item-thresholds, and Fig. 2b shows that the sample is reasonably well targeted by the items as the mean location of the sample is 0.52 (expectation 0) with SD of 1.46 (expectation 1.0).

### Knowledge

The knowledge items are shown in Table 8. Eleven items were tested, all measured on a 5-point ordinal scale of declarative statements related to scientific terminology needed for EBP. Four items had disordered thresholds and needed rescoring. The categories “never heard the term” and “have heard it but don’t understand” were collapsed for items 1, 7, 9, and 10. All 11 items fit the Rasch model after rescoring. Two items showed dependency with at least 1 other item, and the best-worded item was kept resulting in the deletion of items 2 and 11. Three items showed DIF: items 4 and 9 by profession and item 7 by language. In order to include all possible knowledge items, these items were split; however, the DIF by language for item 7 was likely due to poor translation (*English*: *number-needed-to-treat* (*NNT*); *French*: *nombre de sujet à traiter*). The final set of 9 items best reflecting the knowledge all fit the Rasch model and formed a measure (*χ*^2^ = 25.13, df = 18, *p* = 0.12). The threshold map in Fig. 5a shows a hierarchy across knowledge items with the easiest item familiarity with reliability and the hardest item treatment effect size. Figure 5b shows that the people are not well targeted by the items (mean location 2.25, SD 1.45; expectation mean 0, SD 1).

**Table 8:** Results of Rasch analysis for the original items of the construct knowledge of EBP

Instructions: Please indicate your level of agreement with the following statements with respect to your organization or workplace setting: 5-point Likert Scale (Directives: Veuillez indiquer à quel point vous êtes en accord avec les énoncés suivants)						
Item #	Description of item		Response option rescored	Result		
				Item misfit	Local item dependency	DIF
1	E	Reliability of outcome measures	✓	No	No	No
	F	Fidélité de la mesure des résultats				
2	E	Validity of outcome measures		No	Yes with item 10 (item 2 deleted)	No
	F	Validité de la mesure des résultats				
3	E	Sensitivity/specificity of outcome measures		No	No	No
	F	Sensibilité/spécificité de la mesure des résultats				
4	E	Meta-analysis		No	No	Profession (left)
	F	Méta-analyse				
5	E	Confidence interval		No	No	No
	F	Intervalle de confiance				
6	E	Systematic review		No	No	No
	F	Revue systématique				
7	E	Number needed to treat	✓	No	No	Language (item split, French item deleted)
	F	Nombre de sujets à traiter				
8	E	Statistical significance		No	Yes	No
	F	Signification statistique				
9	E	Minimally important change (MIC)	✓	No	No	Profession (left)
	F	Différence minimale cliniquement importante (DMCI)				
10	E	Treatment effect size	✓	No	Yes	No
	F	Ampleur de l’effet du traitement				
11	E	Randomized controlled trial (RCT)		No	Yes with item 8 (item 11 deleted)	No
	F	Essai clinique randomisé				

*DIF* differential item functioning, *E* English, *F* French translation

### Resources

The resources items are shown in Table 9. Fifteen items were tested, all measured on a 5-point Likert Scale for the degree to which the respondent agrees or disagrees with the statement with additional response options for “My work does not involve clinical care” as per the attitudes items. This latter option was merged with the “neutral” option. All 15 items had disordered thresholds and needed rescoring. The categories “strongly disagree” and “disagree” were collapsed for all items. After rescoring, all but 1 item (item 12) fit the Rasch model and it was deleted. Only 1 item showed dependency on another item, and the best-worded item was kept resulting in the deletion of item 14. None of the items showed DIF. The final set of 13 items best reflecting the resources all fit the Rasch model, but the global fit was poor (*χ*^2^ = 56.92, df = 26, *p* = 0.00). Figure 3a shows the hierarchy across the items and Fig. 3b shows that the people are reasonably well targeted by the items, but there are many items at the low end of the resource continuum and no people.

**Table 9:** Results of Rasch analysis for the original items of the construct resources

Instructions: Please indicate your level of agreement with the following statements with respect to your organization or workplace setting: 5-point Likert Scale (Directives: Veuillez indiquer à quel point vous êtes en accord avec les énoncés suivants)						
Item #	Description of item		Response option rescored	Result		
				Item misfit	Local item dependency	DIF
1	E	I am comfortable talking about patient/client care issues with those in charge at the organization	✓	No	No	No
	F	Je suis à l’aise de parler de problèmes liés au soin d’un patient ou client avec les responsables de l’organisation				
2	E	I receive recognition from my manager(s)/supervisor(s) about my work	✓	No	No	No
	F	Mon ou mes supérieurs ou superviseurs apprécient mon travail				
3	E	I have control over how I do my work	✓	No	No	No
	F	J’exerce un contrôle sur *la façon* dont je fais mon travail				
4	E	My organization emphasizes productivity	✓	No	No	No
	F	Mon organisation valorise la productivité				
5	E	My organization supports best practice	✓	No	No	No
	F	Mon organisation soutient les pratiques optimales				
6	E	I have opportunities for educational activities in my organization	✓	No	No	No
	F	J’ai l’occasion d’assister à des activités de perfectionnement dans mon organisation				
7	E	I have formal patient/client related discussions with peers or colleagues (e.g. continuing education, patient rounds, team meetings) in my organization	✓	No	No	No
	F	J’ai des discussions structurées sur les patients ou clients avec mes pairs ou collègues (ex. formation continue, tournée des patients, réunions d’équipe) dans mon organisation				
8	E	I have informal patient/client related discussions with peers or colleagues (e.g. other health care providers, informal bedside teaching) in my organization	✓	No	No	No
	F	J’ai des discussions informelles sur les patients ou clients avec mes pairs ou collègues (ex. autres professionnels de la santé, enseignement au chevet du patient) dans mon organisation				
9	E	My organization routinely provides information/feedback on my practice (e.g. audits, performance reviews)	✓	No	No	No
	F	Je reçois régulièrement de mon organisation une rétroaction ou des commentaires (ex. audit, évaluation de rendement) au sujet de ma pratique				
10	E	I have access to resources at my workplace to help deliver quality care for my patients/clients (e.g. databases, libraries, equipment)	✓	No	No	No
	F	Dans mon milieu de travail, j’ai accès à des ressources qui aident à améliorer la qualité des soins prodigués à mes patients ou clients (ex. bases de données, bibliothèque, équipement).				
11	E	All OT/PT positions at my workplace are currently filled	✓	No	No	No
	F	Tous les postes en physiothérapie ou ergothérapie sont comblés en ce moment dans mon milieu de travail				
12	E	There is a high OT/PT clinician staff turnover rate at my workplace	✓	Yes with a value of 4.41 (item 12 deleted)	No	No
	F	Il y a un fort roulement du personnel clinique d’ergothérapie et de physiothérapie dans mon milieu de travail				
13	E	I have access to space I need to do my job well at my workplace	✓	No	Yes	No
	F	J’ai accès à la place dont j’ai besoin pour bien faire mon travail				
14	E	There is an appropriate space to provide quality care	✓	No	Yes with item 13 (item 14 deleted)	No
	F	Les locaux sont appropriés à la prestation de soins de qualité				
15	E	I have time to do indirect patient activities (e.g. talk about a plan of care, look up something in a journal, get involved in new initiatives at work) in my practice	✓	No	No	No
	F	J’ai le temps de faire des activités indirectement liées aux patients (ex. discuter d’un plan de soin, chercher dans une revue, participer à de nouvelles initiatives professionnelles) dans ma pratique				

*DIF* differential item functioning, *E* English, *F* French translation

## Discussion

This study illustrated the psychometric steps needed to test the extent to which a set of items from existing measures of EBP form measures designed for PTs and OTs. The original 181 items obtained from 5 measures represented the core constructs of EBP. Theoretical and methodological processes were then applied for item reduction which resulted in 68 unique candidate items. Two constructs fit a formative conceptual model: self-use of EBP (9 items) and EBP activities (7 items). Four constructs fit a reflective conceptual model: attitudes towards EBP (*n* = 17 items), self-efficacy (*n* = 9 items), knowledge (*n* = 11 items), and resources (*n* = 15 items).

Rasch analysis showed that items reflecting four core constructs around EBP formed measures: attitudes towards EBP (*n* = 10 items), self-efficacy (*n* = 8 items), knowledge (*n* = 9 items), and resources (*n* = 13 items) (see Table 10). We discuss each one next.

**Table 10:** Summary of the results of the Rasch analysis yielding four measures of EBP

Construct	Items		Thresholds	*N* at ceiling	*p* value for global fit	alpha (*α*)	Threshold range	Item location (SD)	Person location mean (SD)
	Start	Finish							
Attitudes	17	10	35	110 (86%)	0.29	0.63	− 7.51 to 5.04	1.29	− 0.41 (1.41)
Self-efficacy	9	8	22	76 (59%)	0.82	0.80	− 3.70 to 4.91	1.60	− 0.34 (1.12)
Resources	15	13	39	104 (81%)	0.00*	0.86	− 3.38 to 2.86	0.51	− 0.34 (1.73)
Knowledge	11	9	32	108 (84%)	0.12	0.81	− 7.85 to 4.50	1.56	− 0.26 (1.23)
All	52	40							

*All items fit the Rasch model, but improvements are still needed

### Attitudes towards EBP

The items fit the Rasch model but were poorly targeted to our participants primarily because the mean fit for the participants (> 0) suggested that their overall scores on attitudes towards the EBP construct were greater than what would be expected in the general population. This was not surprising as our participants are recent graduates of rehabilitation programmes with a strong focus on EBP. This finding is congruent with previous studies showing recent graduates are more likely to report positive attitudes towards EBP than more seasoned clinicians and those with bachelor’s level training in OT and PT [57–59]. Scale reliability for this construct was unacceptable using person separation index (PSI) = 0.63, indicating that the items inadequately divided our participants along a continuum. RMT was applied to testing attitudes towards EBP in a previous study [27], and the results showed that 10 items fit the Rasch model with good reliability (0.83); however, the authors did not test DIF as the sample size was small (*n* = 110).

Three attitudes items showed DIF: item 2 showed DIF by profession; PTs are more likely than OTs to change their practice because of the evidence they find. Previous research on the use of and attitudes towards EBP showed that OTs score significantly lower than other health care providers which might indicate that this professional group faces different challenges related to EBP compared to other health care providers [60, 61]. Another explanation might be that OT, as a profession, is considered heavily client-centred and as such may value patient preferences as a contributor to EBP more so than the level of evidence [62, 63].

Items 5 and 11 showed DIF by language. Upon review, the English wording was idiomatic and the French translation did not reflect the correct meaning of the idiom. This might be attributed to the lack of proficiency in the English language [64]. The occurrence of DIF in this construct was considered infrequent (2/17 items) and comparable with other findings [65–67]. Future measure development should use simultaneous translation as it has advantages over forward translation particularly in that idiomatic wording; linguistic discrepancies between languages can be minimized or resolved during the item generation process [64, 68].

### Self-efficacy

The final eight items fit the Rasch model and were reasonably well targeted to participants; however, the mean value for the participants was > 0 suggesting that the participants had a higher level of self-efficacy towards EBP than expected from the items. Self-efficacy has good internal reliability (PSI = 0.80), indicating that the items adequately separated the sample along the self-efficacy continuum. The internal consistency reliability value we found is similar to the original source of our items which is the evidence-based practice confidence (EPIC) scale [45]. RMT has been applied to testing self-efficacy towards EBP showing similar findings among different targeted populations with larger sample sizes [31, 32].

### Knowledge

Nine items fit the Rasch model, but the sample was not well targeted by the items with mean fit for the participants (> 0); this suggests that the overall level of knowledge about EBP of our participants was greater than what would be expected from the items. The knowledge construct has a good internal reliability (PSI = 0.81), indicating that the items adequately separated our participants along the measurement continuum. RMT has been used by others [27, 28, 30] to validate knowledge of EBP, and this research has yielded very similar findings to ours.

Three items showed DIF: items 4 which showed that OTs’ familiarity with the statistical term “meta-analysis” is greater than that of PTs’. Item 9 showed that PTs are more aware of the statistical term “minimally important change (MIC)” than OTs. This might be explained by OTs’ use of a combination of different assessment and treatment approaches and their focus on client-centred strategies. Standardized tools are used when relevant and available [69–71]. PTs might know more the measurement-related statistical terms like “minimally important change (MIC)” as they mainly use the standardized tool over qualitative approaches*.* Also, the professional education and exposure to concepts associated with EBP may vary across programmes and between the two professions. Item 7 showed that francophones are more familiar with the term “number-needed-to-treat (NNT)” than anglophones. However, the DIF here was more likely due to the poor translation rather than real differences because “nombre de sujet à traiter” is associated with “caseload” in French than with a statistical term. Harris-Haywood et al. [30] evaluated the measurement properties of the Cultural Competence Health Practitioner Assessment Questionnaire across health care professionals which includes the following domains: knowledge, adapting practice, and promoting health for culturally and linguistically diverse populations. The authors reported DIF by almost all 23 knowledge items: 17 items had DIF by race, 4 by gender, and 6 by profession.

### Resources

The 13 items reflecting resources available for EBP fit the Rasch model and reasonably well for the targeted participants. The mean value was > 0, but there are many items at the low end of the resource continuum with no participants. This suggested that the resources available for our participants were greater than what would be expected from the items included.

This might suggest a selection bias towards people who are good in EBP [72]. Although all items fit, there was still evidence of global misfit, indicating that a review of the items in this measure is warranted. This construct has a good internal reliability (PSI = 0.86), indicating that the items adequately separated participants along the measurement continuum. Bobiak et al. [29] showed almost similar findings in terms of the threshold range of the items and PSI values.

This project would not have been possible without the wealth of items and measures already developed, tested, and used in the study of EBP. However, in order to implement these measures in a practical way, through a survey of EBP of busy practitioners, we had to ensure a minimum number of non-redundant items. Otherwise, the response rate would be nil. This process, which was started for practical reasons, took the field of implementation science a step forward by optimizing the measurement of these important constructs so that it is easier to detect differences between groups and change over time.

Implementation of research in practice is considered to be a multidimensional process that involves clients, practitioners, and organizations [73]. Therefore, the development of measures that can reflect these dimensions is important because it provides tools that help the ultimate goal of implementation for behaviour change (i.e. the use of EBP).

Furthermore, these measures will ease the study of behaviour change mechanisms like those presumed in the Theoretical Domains Framework [74] or Theory of Planned Behaviour [75]. For instance, these mechanisms may include factors such as attitudes towards EBP or knowledge of EBP that in turn impact the intention to use EBP in daily practice. As a result, if we can provide measures of attitudes towards EBP or knowledge of EBP, this then will predict EBP behaviours (i.e. actual use of EBP) [73]. The final set of items provided in this work for each construct fairly covers the full range of the latent variable from “easier” to “difficult” items which will allow researchers to capture different abilities of clinicians regarding aspects of EBP. That by itself will support researchers in the design and implementation of robust knowledge translation interventions in a gradual manner aimed at targeting different levels of clinician abilities instead of designing one-size-fits-all interventions.

#### Strengths

Our study has several strengths. The analyses provide a single legitimate total score for four constructs around EBP on a continuum from very low to very high. These measures can be used to identify determinants of good EBP, to guide the development of implementation interventions, and to evaluate sustainability. The validation of such indices around EBP addresses the lack of standardized tools for EBP educational evaluation [19, 76–78]. This study tackled a specific gap in EBP measures for groups like PTs and OTs in two languages, English and French. The analyses provided a mathematically sound way of reducing the multitude of items to only those that behave in the manner that sufficiently assesses the constructs around EBP while maintaining strong theoretical and methodological processes. We used data from an inception cohort which will allow us to follow participants during the next 3 years to gauge the changes over time. We recruited participants from several Canadian universities in English and French languages which allow for generalizing the findings. The use of simultaneous translation helped to resolve idiomatic wording and linguistic discrepancies between languages compared to sequential translation where items are first generated in only one language followed by subsequent translation into another language [64, 68]. Future work is required to validate these constructs over time, and there is a need to address the gaps in the lower and upper ends of the scales. Further, the correlation between different EBP constructs will allow for greater understanding of the EBP constructs. This will be described in a subsequent descriptive analysis of this large data set and though the ongoing longitudinal study of these graduates into practice.

#### Limitations

Our study has some limitations. First, the data were from a cross-sectional study, and unnecessarily, the data reflect all time points, a weakness reported in previous work [27–32]. Therefore, it is essential to revalidate these constructs with subsequent waves of data to explore stability, intensity, and direction of EBP constructs. Second, the participants were new graduates, and 43% reported that they were not working in clinical practice which limits the use of EBP. Third, the sample size was generally small and diversified across the EBP constructs due to missing data. Fourth, the number of administered items was much less than other questionnaires targeting several constructs; however, it requires about 15 min, which might influence the return rates especially for future time points. Fifth, it was noticed that the level of EBP knowledge for our participants was high which causes a ceiling effect that may explain the high mean person location (> 0) for all EBP constructs. Sixth, despite the fact that the items capture a wide range of difficulty, gaps appear in some locations, making the estimation of the ability of persons located near or within those gaps less precise. Lastly, the knowledge items may not be the true reflection of the participants’ EBP knowledge level since this construct elicits knowledge about statistical and methodological terms, one aspect of EBP. Some items were missed in our knowledge item sets such as knowing how to search literature and different literature appraisal issues.

## Conclusion

This study provides evidence supporting the construct and content validity and internal consistency reliability of four measure to assess key constructs around EBP. Our use of a strong theoretical and methodological processes was helpful in reducing the number of items for further analysis, and the use of Rasch analysis was critical and helpful in choosing the best fitting items for each construct. However, a number of gaps in the measures were uncovered indicating a further refinement of the content and wording is needed.

## Additional file

Additional file 1: A list of included and excluded items. (XLSX 31 kb)

## Abbreviations

DIF: Differential item functioning
EBP: Evidence-based practice
OT: Occupational therapy
PT: Physical therapy
RMT: Rasch Measurement Theory
